# The Role of CXCR3 in the Induction of Primary Biliary Cirrhosis

**DOI:** 10.1155/2011/564062

**Published:** 2011-05-02

**Authors:** Wen Zhang, Yunyun Fei, Jinming Gao, Bin Liu, Fengchun Zhang

**Affiliations:** ^1^Department of Rheumatology, Peking Union Medical College Hospital, Chinese Academy of Medical Sciences, Beijing 100730, China; ^2^Department of Respiratory Disease, Peking Union Medical College Hospital, Chinese Academy of Medical Sciences, Beijing 100730, China; ^3^Department of Rheumatology, Affiliated Hospital of Qingdao University Medical College, 266053, China

## Abstract

*Objective*. Investigate whether CXCR3 and its ligands were involved in the pathogenesis of primary biliary cirrhosis (PBC) in an autoimmune cholangitis animal model. *Methods*. Female C57BL/6 mice were injected with 5 mg/kg of poly I:C intraperitoneally twice a week for 24 weeks. PBC model was confirmed by liver function, serum autoantibodies and liver biopsy. Lymphocytes subsets in liver and spleen and CXCL10 serum level were tested by flow cytometry and ELISA. Liver specimens were collected to evaluate the differences in pathology between WT and CXCR3^−/−^ mice. *Results*. Antimitochondrial antibody was detected in all PBC model. Numbers of infiltrates were detected in the portal areas 8 weeks after poly I:C injection, which progressed up to 24 weeks. Compared to control mice, CXCL10 serum level increased in PBC mice and the proportion of CXCR3^+^ cells increased in the intrahepatic infiltrates of PBC mice, chiefly on CD8^+^ cells, whereas the expression of CXCR3 on CD3^+^ and CD8^+^ splenocytes decreased in PBC model. Compared with WT mice, CXCR3^−/−^ mice developed delayed and milder progression of cellular inflammation. *Conculsions*. CXCR3 might contribute to the development of PBC in murine model. Knockout of CXCR3 might delay and alleviate the PBC disease progression, but could not entirely block the disease development.

## 1. Introduction

Primary biliary cirrhosis (PBC) is an organ-specific autoimmune disease characterized by chronic, progressive destruction of small intrahepatic bile duct with portal inflammation and ultimately fibrosis. Current theories on the pathogenesis of PBC have favored the hypothesis that T lymphocytes played a pivotal role in the autoimmune response in PBC [1, 2]. The liver-infiltrating T cells in PBC might be responsible for damaging the liver and causing chronic liver disease. 

Chemokines played a significant role in regulating the development, differentiation, and location of leukocytes, controlling the migration of immune cells. The chemokine receptors are divided into four subfamilies: CXC, CC, C, CX3C, according to the residual number and arrangement of N-terminal cysteines in their corresponding chemokine ligands. It was reported that CX3CL1 played great role in biliary inflammation in primary biliary cirrhosis [3]. CXCR3, one of the chemokine receptors, was found predominantly on T cells has three different ligands: IFN-inducible protein 10 (IP-10)/CXCL10, monokine induced by IFN-*γ* (Mig)/CXCL9, and IFN-inducible T cell *α* chemoattractant (ITAC)/CXCL11 [4–6], playing a critical role in the recruitment of T cells to inflammatory sites and regulating T cell activation [7].

The expression of CXCR3 has been reported to play a crucial role in development of several immunological disease states (e.g., systemic lupus erythematosus, multiple sclerosis, rheumatoid arthritis, psoriasis, dermatomyositis, and inflammatory bowel disease) [8–18]. In addition, it was reported that CXCR3 and its ligands contributed to the development of necroinflammatory liver damage and progression towards fibrosis in chronic HCV patients [19–28] and other virus-infected liver diseases [29, 30], which implied the possible involvement of chemokine (IP-10 and MIG)-chemokine receptor (CXCR3) interactions in the pathogenesis of PBC [31, 32]. Although studies in human PBC have shown the presence of CXCR3^+^ T cells in liver tissues, the contribution of CXCR3 to the recruitment of T cells to these inflamed tissues was not well established. Moreover, it was unclear whether CXCR3 was essential for migration to this inflamed tissue in PBC.

In this study, we hypothesized that CXCR3 played important role in T cell migration to sites of inflammation in PBC, and CXCR3 gene deficiency would terminate or attenuate the severity of PBC. To address this hypothesis, we used CXCR3 knockout (CXCR3^−/−^) mice and their wild-type (WT) littermates to investigate the contribution of CXCR3 in PBC.

## 2. Materials and Methods

### 2.1. Mice

Four groups of C57BL/6 background mice, 10 to 12 weeks old (20–22 grams of weight), 24 in each group, were used for this experiment: Group I, WT control mice, which were injected intraperitoneally with sterilized phosphate-buffered saline (PBS); Group II, CXCR3^−/−^ control mice, which were injected with PBS; Group III, WT PBC mice, which were injected with Polyinosinic polycytidylic acid (Poly I:C) and Group IV, CXCR3^−/−^ PBC mice, which were injected with poly I:C. The mice were sacrificed by cervical dislocation at weeks 8, 16, and 24, respectively, after administration of poly I:C or PBS. Eight mice were sacrificed in every group at different time point. Three mice had died before test time point. CXCR3^−/−^ mice with C57BL/6 background had been established by gene targeting as described elsewhere and were kindly provided by Dr. Bao LU (Harvard Medical School, Boston, Mass, USA). CXCR3^−/−^ mice and WT littermate mice (Experimental Animal Research Center, Beijing, China) with C57BL/6 background (backcross more than 14 generations) were maintained in a pathogen-free Mjplouse facility at Peking Union Medical College (Beijing, China).

### 2.2. Poly I:C Injection

Poly I:C was obtained from Invivogen. It was dissolved in sterilized water at a concentration of 1 mg/mL and stored at −20°C until needed. Female C57BL/6 mice were injected with poly I:C (1 mg/kg of body weight) intraperitoneally twice a week for 24 consecutive weeks. As controls, a group of female C57BL/6 mice was injected with PBS.

### 2.3. Preparation of Splenocytes and Liver-Infiltrating Lymphocytes

Lymphocytes were isolated from livers or spleens using nonenzymatic, mechanical methods to preserve chemokine receptor expression. After mice were sacrificed, the abdomen was opened, and the portal vein was cut to create an outlet. The caval vein was then cannulated and perfused with 5 to 10 mL of PBS. Perfused livers and spleens were homogenized in 20 mL of Hank's balanced salt solution (HBSS) by passing the tissue through a fine nylon mesh, and cells were pelleted by centrifugation at 500 ×g. Pellets were resuspended by PBS and added over Ficoll density gradients. Mononuclear cells were then pelleted by 20 min of centrifugation at 1000 ×g. Isolated lymphocytes were resuspended in RPMI 1640.

### 2.4. Flow Cytometric Analysis

Splenocytes and liver-infiltrating lymphocytes were prepared by centrifugation over Ficoll density gradients and were incubated with phycoerythrin- (PE-) conjugated anti-CXCR3, Fluorescein isothiocyanate- (FITC-) conjugated anti-CD4 or anti-CD8, or Phycoerythrin-Cy5- (PE-Cy5-) conjugated anti-CD3, which were purchased from eBioscience company. Flow cytometric analysis was performed on FACScan flow cytometer (Coulter EPICS XL).

### 2.5. Detection of Autoantibodies

Two methods were employed to detect autoantibodies in the mice serum. The existence of autoantibodies, including antinuclear antibody (ANA), antimitochondrial antibody (AMA), and antismooth muscle antibody (SMA), was firstly examined by staining Hep (Human epidermoid cancer cells)-2 cells using indirect immunofluorescent technique. The Euroimmun anti-M2-3E ELISA (IgG) test kit provides a semiquantitative in vitro assay for autoantibodies of the immunoglobulin class against the mitochondrial antigens M2 in serum according to the manufacture's protocol. Horseradish peroxidase- (HRP-) conjugated affinity purified goat antimouse IgG (Jackson ImmunoResearch Laboratories) was used as a secondary antibody. Photometric measurement was made at a wavelength of 450 nm.

### 2.6. IP-10/CXCL10 ELISA

ELISA kit specific to murine IP-10 was purchased from R&D Systems. This assay employed the quantitative sandwich enzyme immunoassay technique. A polyclonal antibody specific for mouse IP-10 had been pre-coated onto a microplate. Standards, controls, and samples were pipetted into the wells. The concentrations of CXCL10 in the sera were determined by ELISA kits according to the manufacturer's recommendations.

### 2.7. Histopathology and Immunohistochemical Examination

Formalin-fixed, paraffin-embedded tissues were used for histological evaluation. After deparaffinization, various tissues were stained with hematoxylin and eosin (H&E staining). The extent of infiltrating cells in the liver tissues was evaluated by light microscopy. Expression of CXCR3, CD4 and CD8 was detected by immunohistochemistry using rabbit anti-mouse CXCR3, anti-mouse CD4, and anti-mouse CD8 (Beijing Biosynthesis Biotechnology CO, LTD) diluted 1 : 1000, respectively.

### 2.8. Statistical Analysis

Data were expressed as the mean value ± SEM of the number of samples evaluated. Student's *t*-test or ANOVA was used to evaluate the significance of the differences. Statistical analysis was performed with SPSS version 11.5, with a value of *P* less than  .05 regarded as statistically significant.

## 3. Results

### 3.1. Clinical Features of Female C57BL/6 Mice Injected with Poly I:C

Liver function tests were performed in all the control and PBC model mice. Serum alanine aminotransferase (ALT) and alkaline phosphatase (ALP) levels were raised among WT PBC mice compared with WT control mice (48.45 ± 24.69 IU/L versus 27.00 ± 7.78 IU/L, *P* = .043; 117.74 ± 38.02 IU/L versus 71.17 ± 8.61 IU/L, *P* = .005), as well as serum total bilirubin (TBIL) levels (1.89 ± 1.12 *μ*mol/L versus 0.24 ± 0.12 *μ*mol/L, *P* = .001). Serum levels of ALT, ALP, and TBIL of CXCR3^−/−^ PBC mice were 46.70 ± 19.18, 108.40 ± 28.08 IU/L, and 1.68 ± 1.04 *μ*mol/L respectively. Although ALT levels were found to be elevated in all PBC model mice regardless of genotype compared to CXCR3^−/−^ PBC mice, WT PBC mice had higher TBIL level in the serum, but with no statistical significance (2.08 ± 1.19 *μ*mol/L versus 1.68 ± 1.04 *μ*mol/L), as well as ALP level (117.72 ± 38.54 IU/L versus 108.40 ± 28.08 IU/L).

### 3.2. Development of Autoantibodies in Female C57BL/6 Mice Injected with Poly I:C

**Figure 1:**
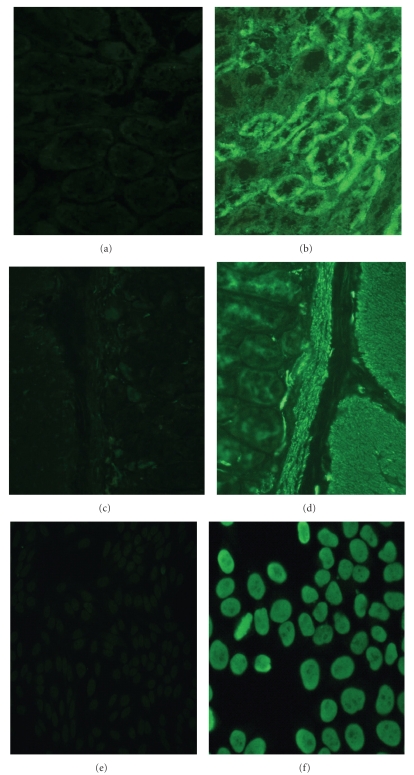
A representative staining pattern of autoantibodies from the sera of female C57BL/6 mice injected with poly I:C and control mice. No autoantibody was detected in the sera from control mice (a, c, e). The presence of anti-mitochondrial antibody (b), anti-smooth muscle antibody, (d) and antinuclear antibody (f) was evaluated by immunofluorescence method.

Autoantibodies were detected in all experimental mice. As shown in Figure 1, a variety of autoantibodies were detected in the sera from poly I:C-injected mice, including AMA, ANA, and SMA. By 24 weeks, all of the poly I:C-injected mice exhibited more than one type of autoantibodies (Table 1). 

**Figure 2:**
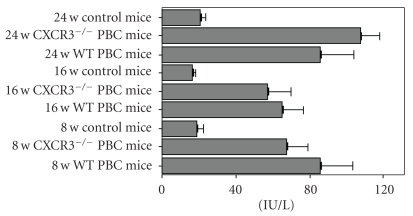
AMA titers in the control and PBC mice sera measured by ELISA.

Serum AMA was measured by ELISA (Figure 2). AMA titers were increased in the WT PBC mice compared with WT control mice (78.69 ± 39.04 versus 18.82 ± 9.06 IU/L, *P* = .001). There was no significant difference between AMA titers in the serum of WT PBC mice and CXCR3^−/−^ PBC mice (78.69 ± 39.04 versus 80.14 ± 39.27 IU/L) or between different time points (data not shown). 

### 3.3. Surface Expression of CXCR3 on CD3^+^, CD4^+^, and CD8^+^ Cells

**Figure 3:**
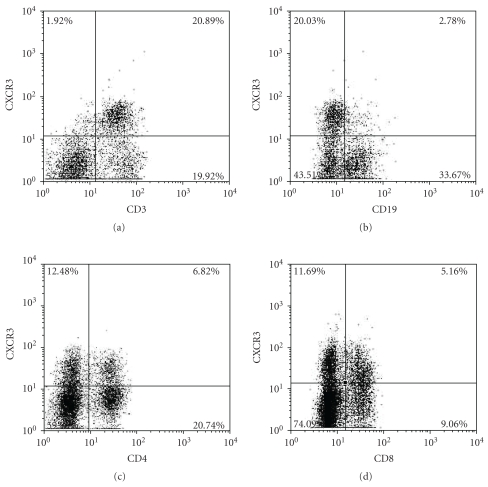
A representative data showed surface expression of CXCR3 on CD3^+^, CD19^+^, CD4^+^, and CD8^+^ cells on splenocytes from WT control mice.

Splenocytes and liver-infiltrating lymphocytes of eighteen WT mice including nine control mice and nine PBC model mice were tested by flow cytometry. To analyze the expression of CXCR3 on lymphocytes, splenocytes were stained from WT control mice with anti-CD4, anti-CD8, anti-CD3, anti-CD19, and anti-CXCR3 mAb. A representative result of the flow cytometry analysis was shown in Figure 3. CXCR3 were expressed predominantly on CD3^+^ cells, rather than CD19^+^ cells. The percentage of CXCR3^+^ cells was higher in the CD8^+^ T cell population than that in the CD4^+^ cells (Figure 3). 

**Figure 4:**
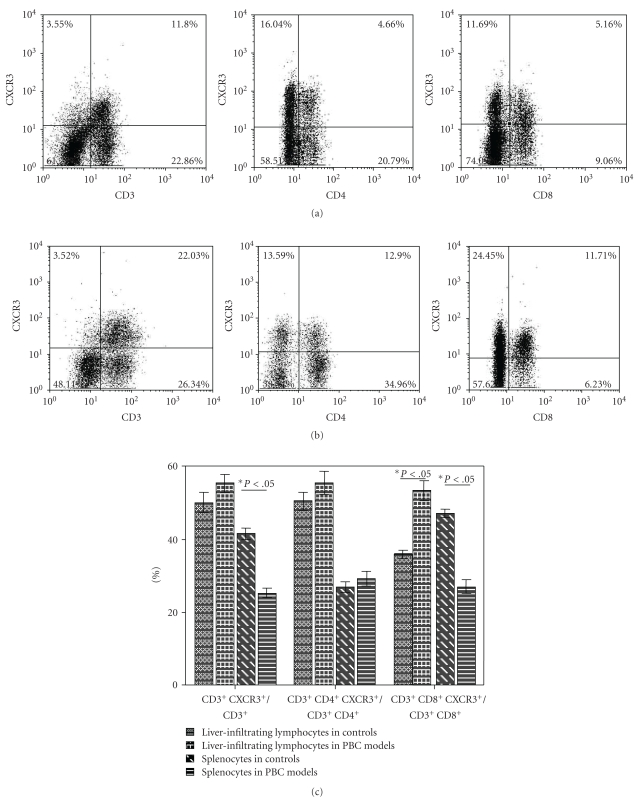
Expression of CXCR3 on T cells in control and PBC mice. (a) Expression of CXCR3 on CD3^+^, CD4^+^, and CD8^+^ intrahepatic lymphocytes in the control mice. (b) Expression of CXCR3 on CD3^+^, CD4^+^, and CD8^+^ intrahepatic lymphocytes in WT PBC mice. (c) Comparison between expression of CXCR3 on CD3^+^CD4^+^, and CD3^+^CD8^+^ lymphocytes in WT control and PBC mice. Data are depicted as the mean value ± SEM of percentages. Statistical significance is determined by the one-tailed paired two-sample Student's *t*-test and is indicated as follows: *P < .05.

To determine whether distinct subsets of peripheral immune cells are selectively recruited to the liver, we examined the expression of CXCR3 on liver-infiltrating lymphocytes at week 8, 16 and 24 (Figure 4) by flow cytometry which demonstrated a significantly higher proportion of CXCR3 expressed on CD3^+^, CD4^+^ and CD8^+^ cells in liver than in spleen, especially in the WT PBC model mice. Compared to control model, the proportion of cells positive for CXCR3 was increased in the intrahepatic infiltrates of WT PBC mice, with significant difference on CD8^+^ T cells (*P* = .039) (Figures 4(a), 4(b), and 4(c)). Furthermore, the proportion of CD3^+^ and CD8^+^ splenocytes positive for CXCR3 was significantly higher in WT control mice than in WT PBC mice (*P* = .002 and *P* = .019) (Figure 4(c)). 

High percentage of CXCR3^+^ cells in the liver suggested that CXCR3^+^ lymphocytes were recruited to the liver through specific mechanisms. The high expression of CXCR3 might be an important event in migration of T cells to sites of inflammation.

### 3.4. Analysis of IP-10/CXCL10 in the Mice Sera

The serum level of IP-10/CXCL10 in all PBC and control mice was analyzed. Significantly increased serum levels of IP-10/CXCL10 were observed in WT PBC models as compared with control subjects (0.38 ± 0.25 versus 0.09 ± 0.04, *P* = .002). With the disease progression, IP-10/CXCL10 level was elevated gradually. Furthermore, during 8-weeks and 16-weeks periods, IP-10/CXCL10 increased slightly in CXCR3^−/−^ PBC mice without statistical significance compared with WT PBC mice (0.39 ± 0.18 versus 0.28 ± 0.10 at week 8 and 0.46 ± 0.45 versus 0.33 ± 0.19 at week 16). In 24-weeks period, IP-10/CXCL10 serum level increased significantly in CXCR3^−/−^ PBC mice compared with WT PBC mice model (0.51 ± 0.23 versus 0.27 ± 0.11, *P* = .046).

### 3.5. Histological Features of the PBC Model Liver

**Figure 5:**
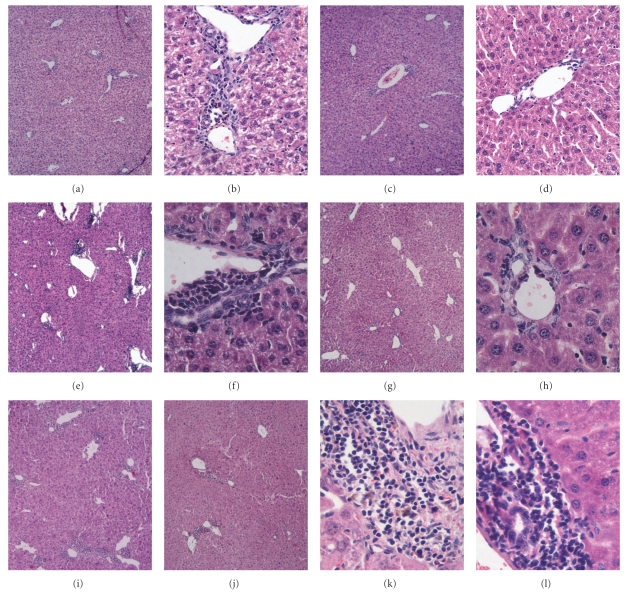
Serial observation of mononuclear cells infiltration in the liver tissue. Female C57BL/6 mice were injected with 5 mg/kg of poly I:C twice a week for 24 consecutive weeks. Liver specimens were collected at 8 weeks (a–d), 16 weeks (e–h), and 24 weeks (i–l) after the start of poly I:C injection and were underwent HE staining. Different degrees of lymphocytic infiltration surrounding the small bile ducts were detected within the portal tracts. A representative staining pattern from each period is shown as following: (a, b) Week 8 of WT PBC mice. (c, d) Week 8 of CXCR3^−/−^ PBC mice. (e, f) Week 16 of WT PBC mice. (g, h) Week 16 of CXCR3^−/−^ PBC mice. (I, k) Week 24 of WT PBC mice. (j, l) Week 24 of CXCR3^−/−^ PBC mice. *Note*: Magnification: (a, c, e, g, i, j) × 5; (b, d, f, h, k, l) × 20, × 40.

After injection with 5 mg/kg of poly I:C, different degrees of lymphocytic infiltration surrounding the small bile ducts were detected within the portal tracts (HE staining) in WT C57BL/6 mice. Representative staining patterns of mononuclear cell infiltration in the liver tissues are shown in Figure 5. Mononuclear cell infiltration in the liver tissues was detected at 8 weeks after poly I:C injection Figures 5(a), and 5(b) and progressively increased with time. A representative staining pattern of portal areas at 16 weeks after the start of poly I:C injection is shown in Figures 5(e), 5(f), which reveals irregular nuclear arrangement of interlobular bile ducts, distorted lumen and detachment of cells, as well as infiltrating cells accumulated around the damaged bile duct, resembling chronic nonsuppurative destructive cholangitis as seen in PBC. In 24-week period, the interlobular bile ducts were lost, bileplugs were evident in the livers of WT PBC mice, and granulomas formation was detected as well (Figures 5(i), and 5(k)). Furthermore there was a mild interface hepatitis (piecemeal necrosis), and, in a few instances, infiltrates were evident adjacent to the central vein.

By comparing histological changes of CXCR3^−/−^ PBC mice with WT PBC mice, we analyzed hepatic inflammation and granuloma's formation in the livers of PBC models of WT and CXCR3^−/−^ mice. The results showed there was significant reduction of inflammation foci and inflammatory cells in the livers of CXCR3^−/−^ PBC mice than that in WT PBC mice at weeks 8, 16. Furthermore, at week 24, there was no cholestasis or granulomas in CXCR3^−/−^ PBC mice. These findings indicated that CXCR3^−/−^ mice had a delayed onset and insufficient recruitment of inflammatory cells to the liver. 

### 3.6. Immunohistochemical Examination Results

**Figure 6:**
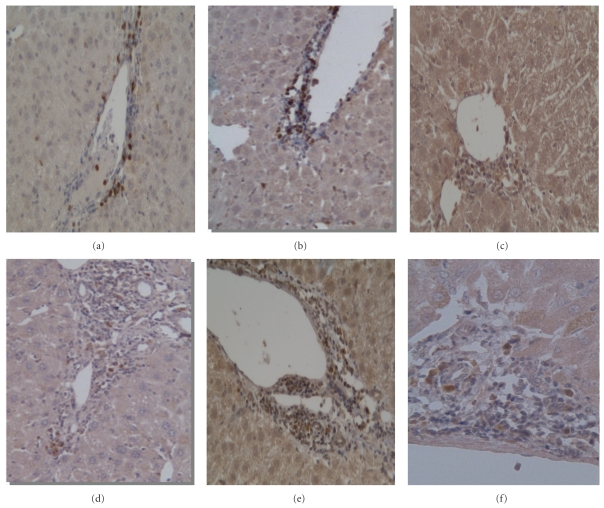
Immunohistochemical staining of liver from PBC model mice. Representative results of immunohistochemical staining of CD4, CD8, and CXCR3 in the early and late stage of PBC were shown as follows (×20 magnifications): (a) CD4^+^ lymphocytes at week 8; (b) CD8^+^ lymphocytes at week 8; (c) CXCR3^+^ lymphocytes at week 8; (d) CD4^+^ lymphocytes at week 24; (e) CD8^+^ lymphocytes at week 24; (f) CXCR3^+^ lymphocytes at week 24.

As shown in Figure 6, immunohistochemical analysis of liver consistently confirmed that, in PBC model mice, there were numerous of CD4^+^ and CD8^+^ lymphocytes infiltrated in portal area which was absent in control mice. The distribution of CD4^+^   CXCR3^+^ and CD8^+^ CXCR3^+^ cells was similar. Moreover, CXCR3^+^ mononuclear cells were found mainly in the enlarged portal tracts, localized near the bile ducts, especially along the periportal areas (Figure 6(f)). Few CD4^+^ or CD8^+^ T cells were seen in hepatic lobules.

## 4. Discussion

CXCR3 is a G protein-coupled, seven-transmembrane receptor found predominantly on T cells that binds and is activated by the three IFN-gamma-inducible chemokines of the CXC family named CXCL9, CXCL10, and CXCL11 [4, 5]. Their major function is to selectively recruit immune cells at inflammation sites, but they also play a role in angiogenesis mechanisms [33]. In the last few years, strong experimental and clinical evidence has been obtained supporting the idea that the CXCR3 pathway is involved in the development of autoimmune diseases, thereby inducing worsening of clinical manifestations [8–17]. CXCL10 is a 10 kD protein, which is categorized functionally as a Th1-chemokine. It binds to the receptor CXCR3 and regulates immune responses through the activation and recruitment of leukocytes [6]. Recent reports have shown that serum and/or tissue expressions of CXCR3 and CXCL10 are increased in various autoimmune diseases, which may have important roles in leukocyte homing to inflamed tissues and in the perpetuation of inflammation, and therefore, tissue damage [5, 6]. Although previous studies in human PBC have shown the presence of CXCR3^+^ T cells in liver tissues, the contribution of CXCR3 to the recruitment of T cells to these inflamed tissues is not well established [31]. Also, it is unclear whether CXCR3 is essential for migration to this inflamed tissue. 

 In this study, WT C57BL/6 mice and CXCR3^−/−^ C57BL/6 mice were used to establish PBC mice model, which had been demonstrated by Okada et al. [34]. We investigated whether CXCR3 was involved in the pathogenesis of PBC, by methods of IIF, ELISA, pathology, immunohistochemistry, and flow cytometry. 

First, we analyzed the pathologic character of WT mice injected with poly I:C. In our study, moderate to severe lymphocytes infiltration was detected within the portal tracts in association with bile duct damage, occurring only in the mice injected with poly I:C, resembling those of chronic nonsuppurative destructive cholangitis as seen in PBC. Moreover, it was difficult to identify an intact bile duct structure because biliary cell destruction was sufficiently advanced in liver tissues from the poly I:C-injected mice. As illustrated in sections of liver, portal tracts of livers of PBC model mice contained moderate infiltrates of mononuclear cells associated with biliary ductular damage. Interlobular bile ducts showed an irregular nuclear arrangement, a distorted lumen and detachment of cells from the basement membrane, similar to that seen in advanced human PBC. 

Secondly, we analyzed the serum AMA level in control mice, WT mice, and CXCR3^−/−^ mice which were injected with poly I:C. In human PBC, the severity and temporal progression vary widely among patients and do not correlate with AMA titers. In this study, a variety of autoantibodies were detected positive in the sera from poly I:C-injected mice, including AMA, ANA, and SMA. The results suggested that the poly I:C-injected mice not only had the inflammatory infiltration around portal tracts, but also showed some character of autoimmune hepatitis. In addition, there was no significant difference between AMA titers in the serum of WT mice and CXCR3^−/−^ mice, or between different period of PBC, which revealed that the disease severity varied widely among different period mice injected with poly I:C, but did not correlate with AMA titers, both in WT mice and CXCR3^−/−^ mice. 

Next, the functional importance of CXCR3 in the liver of PBC mice model was investigated by flow cytometry, which confirmed the selective recruitment of CXCR3^+^ lymphocytes into the liver. We found the increased expression of CXCR3 in the liver after poly I:C injection as well as its ligands IP-10/CXCL10 in the serum. We analyzed paired samples of liver infiltrating lymphocytes and splenocytes from PBC model mice and control mice and detected the expression of CXCR3 in the liver there was greater expression of CXCR3 than that from spleen. Compared to control model, the proportion of cells positive for CXCR3 was increased in the intrahepatic infiltrates in PBC model, chiefly on CD8^+^ T cells; furthermore, the proportion of CXCR3^+^ cells expressed on CD3^+^ and CD8^+^ cells was markedly decreased in the splenocytes in PBC model. The preferential accumulation of CXCR3^+^ cells in liver and increased level of its ligand IP-10/CXCL10 in serum suggested that CXCR3 expressing cells migrated to the liver in a much larger number than T cells lacking this chemokine receptor. Therefore, CXCR3 might play a great role in recruiting T cells, especially CD8^+^ T cells to liver tissues in PBC disease model. In addition, using immunohistochemistry we demonstrated that all the PBC models showed CXCR3-expressing lymphocytes infiltrating the liver. 

Forth, importantly, we directly demonstrated an important role for CXCR3 in inflammatory cells recruitment to inflamed liver in PBC by comparing the pathologic result of WT mice, and CXCR3^−/−^ mice. Although the statistically significant decrease of liver function test, including ALP and TBIL, was not achieved in CXCR3^−/−^ PBC mice compared with WT PBC mice, the histopathological results confirmed the effect of CXCR3 to the recruitment of inflammatory cells in PBC model disease development. In the WT mice injected with poly I:C, moderate to severe lymphoid cell infiltration was detected within the portal tracts in association with bile duct damage. Compared to WT mice, although the same titer of AMA was produced in CXCR3^−/−^ mice injected with poly I:C, there was marked reduction in total inflammatory cells in the liver over 8- and 16- week periods, leading to insufficient inflammatory cells infiltration. Moreover, cholestasis and fibrosis were not detected in PBC model of CXCR3^−/−^ mice in 24 weeks. These findings provided direct evidence that CXCR3 could mediate inflammatory cells recruitment to inflamed tissues in liver and, hence, had a major role in inflammation of PBC model mice. Nevertheless, knockout CXCR3 could reduce the severity of the disease but not entirely eliminate inflammatory cells infiltration in the liver. This observation strongly suggested that CXCR3 on T cells might play an important but not absolutely essential role in T cells recruitment to inflamed liver tissues and other CXCR3-independent pathways also mediated recruitment in the disease development. 

 The limit of this study is that there were a variety of autoantibodies tested in the PBC mice model, including AMA, ANA, and SMA, indicating some character of autoimmune hepatitis and other sites of inflammation. Salivatitis, pancreatitis, and interstitial nephritis were demonstrated in the article of Okada et al. [34]. Further studies with more specific PBC models are needed. 

In conclusion, this study directly demonstrated that chemokine IP-10 and its receptor CXCR3 promote the action of lymphocytes in PBC model and that CXCR3 knockout dramatically reduces the severity and inflammatory reaction in PBC. Given the role that CXCR3 and its ligands play in induction of hepatic damage, prevention of CXCR3 engagement may be beneficial for the treatment during the lymphocytes infiltration period.

## Figures and Tables

**Table 1:** The serum autoantibodies detected by indirect immunofluorescence in PBC mice.

Type of Abs	WT PBC mice Positive/total number			CXCR3^−/−^ PBC mice Positive/total number		
	8 weeks	16 weeks	24 weeks	8 weeks	16 weeks	24 weeks
ANA	3/8	5/8	5/7*	2/8	4/7*	5/7*
AMA	3/8	6/8	7/7*	4/8	5/7*	6/7*
SMA	1/8	2/8	1/7*	0/8	1/7*	2/7*

*Every group was consisted of 8 mice. There were seven mice because 1 mouse had died before test.
